# Possible Seasonality of *Clostridium difficile* in Retail Meat, Canada

**DOI:** 10.3201/eid1505.081084

**Published:** 2009-05

**Authors:** Alexander Rodriguez-Palacios, Richard J. Reid-Smith, Henry R. Staempfli, Danielle Daignault, Nicol Janecko, Brent P. Avery, Hayley Martin, Angela D. Thomspon, L. Clifford McDonald, Brandi Limbago, J. Scott Weese

**Affiliations:** University of Guelph, Guelph, Ontario, Canada (A. Rodriguez-Palacios, R.J. Reid-Smith, H.R. Staempfli, N. Janecko, H. Martin, J.S. Weese); Public Health Agency of Canada, Guelph (R.J. Reid-Smith, B.P. Avery); Public Health Agency of Canada, Saint-Hyacinthe, Québec, Canada (D. Diagnault); Centers for Disease Control and Prevention, Atlanta, Georgia, USA (A.D. Thompson, L.C. McDonald, B. Limbago)

**Keywords:** Enteric infections, bacteria, Clostridium difficile, foodborne, ribotypes, seasonality, hypervirulent, meat, Canada, dispatch

## Abstract

We previously reported *Clostridium difficile* in 20% of retail meat in Canada, which raised concerns about potential foodborne transmissibility. Here, we studied the genetic diversity of *C. difficile* in retail meats, using a broad Canadian sampling infrastructure and 3 culture methods. We found 6.1% prevalence and indications of possible seasonality (highest prevalence in winter).

*Clostridium difficile* infection (CDI) has been associated with increased illness and death in Canada since 2000 (*1*,*2*). Although multiple genotypes with higher levels of virulence and antimicrobial resistance have been recognized (*1*,*3*), little is known about risk factors for CDI acquisition outside healthcare facilities.

In a 2005 study, we found *C. difficile* in 20% of retail meats sampled in Canada (*4*). Limitations to that study included limited geographic representation, nonsystematic sampling, and the use of a nonvalidated culture method. These sampling limitations prevent valid extrapolations. Broader sampling and a better understanding of the culture methods were thus required to reassess the prevalence of retail meat contamination with *C. difficile*. Here, we determined the prevalence of *C. difficile* in retail meat by using a broad-based government sampling infrastructure, compared 3 culture methods, characterized recovered isolates, and evaluated month-to-month variability in *C. difficile* recovery.

## The Study

Retail meats were obtained from 2 randomly selected census divisions per week from various retailers across Canada as part of the active retail surveillance component of the Canadian Integrated Program for Antimicrobial Resistance Surveillance (CIPARS) (*5*). We tested random packages of ground beef as well as veal chops from milk-fed calves; the packages were purchased by CIPARS in Ontario, Québec, and Saskatchewan, Canada, from January through August 2006. Purchased packages were sent to the Laboratory of Foodborne Zoonoses, Québec (ground beef), and to the Canadian Research Institute for Food Safety, Ontario (veal chops), where 35-g composite samples were made. Rinsates were prepared by mixing 25 g of meat and 225 mL of buffered peptone water (placed in a stomacher for 15 min). Rinsates (12 mL) and the remains of the composite samples (10 g) were then sent to the University of Guelph for *C. difficile* testing. Sample size estimations indicated that 211 packages were adequate to verify a prevalence of 20% ± 8% (α = 0.05, power = 0.8; Stata sampsi command [Stata Corp., College Station, TX, USA]).

A total of 214 meat samples were cultured by using 3 methods. One method, used in an earlier study (*4*), was tested in duplicate to assess reproducibility. All protocols had an enrichment phase of 7 days (Table 1), followed by ethanol treatment of culture sediments (96%, 1:2 [vol/vol], 30 min), and inoculation onto solid agar for colony identification (*4*,*6*).

**Table 1:** Proportion of retail meat packages yielding *Clostridium difficile* in 4 culture replicates and estimated method sensitivity, Canada, 2006*†

Sample	Culture method			% Samples with *C. difficile*			Culture sensitivity, %‡
	Enrichment	Agar		Ground beef	Veal from milk-fed calves	Both‡	
Rinsate	TCDMNB	CDMNA		2.7 (4/149)§	0 (0/65)	1.9 (4/214)	31
Meat¶	TCDMNB	CDMNA		2.7 (4/149)§	1.5 (1/65)	2.3 (5/214)	39
Meat¶	TCDMNB	CDMNA		1.3 (2/149)§	1.5 (1/65)	1.4 (3/214)	23
Meat	TCCFB	Blood		1.3% (2/149)	1.5 (1/65)	1.4 (3/214)	23
Total of contaminated packages#				6.7 (10/149)	4.6 (3/65)	6.1 (13/214)‡	100

*Rinsate, sediment; TCDMNB, in-house *C. difficile* broth (CM0601; Oxoid, Basingstoke, UK) supplemented with cysteine hydrochloride, moxalactam, norfloxacin (CDMN, SR0173E; Oxoid), and 0.1% sodium taurocholate (Sigma-Aldrich, Inc., St. Louis, MO, USA) (*4*); Meat, 2 g; CDMNA, *C. difficile* agar supplemented with CDMN and 7% laked horse blood (SR0048C; Oxoid); TCCFB, broth supplemented with D-cycloserine and cefoxitin (SR0096E; Oxoid) and 0.1% sodium taurocholate; Blood, 5% defibrinated sheep blood. †Poor test agreement was found among and between cultures (κ –0.28; p>0.9). ‡Culture sensitivity calculation based on parallel interpretation of all 4 cultures (standard comparator) and 6.1% of overall contamination. Duplicate testing sensitivity ranged from 46.2% (6/13) to 61.5% (8/13). §Represents 2 packages that simultaneously tested positive in 2 culture replicates. ¶Protocol previously used to test meat; duplicate run (*4*). #No statistical differences were found between ground beef and veal in any culture replicate (p>0.1).

Suspected colonies (swarming, nonhemolytic) were subcultured onto 5% sheep blood agar. *C. difficile* was preliminarily identified with L-proline aminopeptidase activity (Pro Disc; Remel, Lenexa, KS, USA) but confirmed by PCR detection of the triose phosphate isomerase gene (*7*).

PCR ribotyping and detection of genes for toxins A (*tcdA*), B (*tcdB*), binary toxin (*cdtB*), and toxin regulator (*tcdC*) were performed as previously described (*4*,*8*,*9*). Isolates having either *tcdA*, *tcdB*, or *cdtB* were classified as toxigenic (*10*).

Resulting PCR ribotypes were visually compared to representative PCR ribotypes previously identified in cattle (n = 8, 2004), retail meats (n = 4, 2005), and humans (n = 39, 2004–2006) in Ontario and Québec, Canada (*2*,*4*,*11*). The first meat-derived isolate of each PCR ribotype and 1 matching human isolate were submitted to the Centers for Disease Control and Prevention, Atlanta, Georgia, USA, for *Sma*I pulsed-field gel electrophoresis (PFGE) and toxinotyping (*1*).

We tested selected isolates to determine the MICs of clindamycin, levofloxacin, moxifloxacin, and gatifloxacin by using the Etest (AB Biodisk, Solna, Sweden) and interpreted the results after the isolates were incubated for 48 h on *Brucella* agar (*12*). Controls included *C. difficile* strain ATCC 700057.

Culture binary data were analyzed by using a randomized block design approach with a conditional logistic regression analysis (SAS Institute, Cary, NC, USA) and p value estimations with Monte Carlo simulations. Exact tests for pairwise comparisons were based on LogXact 7 and a Fortran program (Cytel Inc, Cambridge, MA, USA). Kappa, χ^2^, and Fisher exact tests were also used. Significance was held at p<0.05.

**Figure 1:**
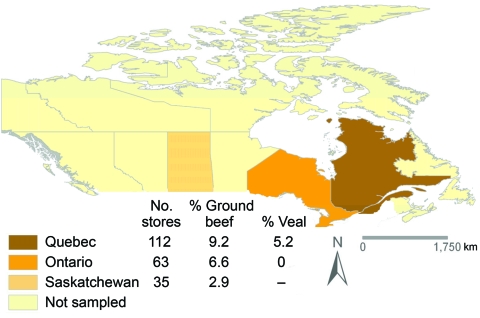
Distribution of retail grocery stores sampled (n = 210) and proportion with contaminated meat. The overall proportion of stores with >1 meat package contaminated with *Clostridium difficile* was 5.7%. No statistical differences were observed when comparing the proportions of ground beef contamination in Québec, Ontario, and Saskatchewan, Canada (p>0.2). No comparisons for veal chops were made because Québec was the main source of this commodity; veal from milk-fed calves was not available in Saskatchewan, and only 3 stores had this type of veal during sampling in Ontario.

In total, 149 ground beef and 65 veal chop samples, obtained from 210 retailers in Canada, were cultured for *C. difficile* (Figure 1). The numbers of samples tested per month were 12, 49, 34, 5, 73, 31, 0, and 7, from January through August in 2006; 3 samples lacked sampling dates.

Combining the results from 4 cultures, we found the prevalence of *C. difficile* was 6.7% (10/149) in ground beef and 4.6% (3/65) in veal chops from milk-fed calves. The combined prevalence was 6.1% (13/214). The prevalence of *C. difficile* recovery determined by using different culture methods varied from 1.4% to 2.3%, but no culture agreement or reproducibility was observed (p>0.1). Overall, the individual diagnostic sensitivity of each method was low (<39%; Table 1).

When month-to-month variability was considered, *C. difficile* was more commonly isolated from meat in January and February (11.5%, 7/61) than during the remaining 5 months of the study (4%; 6/150; p = 0.041). This finding indicates possible seasonality, although further studies are needed.

A total of 28 *C. difficile* isolates were cultured from 13 meat packages (22 from ground beef; 6 from veal). PCR ribotyping showed 8 distinct genotypes, 7 of which were toxigenic and present in 10 (77%) meat packages (Table 2). Genotypes resembling human PCR ribotype 027/NAP1 were found in 30.8% (n = 4) of positive samples, and PCR ribotypes 077/NAP2 and 014/NAP4, formerly reported in cattle and retail meats (*3*,*4*), were identified in 23.1% (n = 3) and 15.4% (n = 2) of samples, respectively. Multiple genotype contamination was also documented (2 PCR ribotypes/sample, n = 2).

**Table 2:** Molecular characteristics of 15 representative *Clostridium difficile* strains isolated from 13 of 214 retail meat packages tested in Canada, 2006*

Type†	% (no.)	Toxin genes‡	*tcdC* deletion	Toxinotype	PFGE§	Product–culture	Month	Province
M26	23.1 (3)	A^–^B^–^, *cdtB^–^*	NA	Nontypeable	Unnamed	VC–C3	Feb	QC
					–	GB–C2	Jan	ON
					–	GB–C2	Jun	SK
077¶	23.1 (3)	A^+^B^+^, *cdtB^–^*	No	0	NAP2	GB–C3	Jan	QC
					–	GB–C1	Jan	ON
					–	GB–C3	Jan	QC
J¶	23.1 (3)	A^+^B^+^, *cdtB*^+^	18 bp	III	NAP1	GB–C4	May	ON
					NAP1a	GB–C4	Jun	ON
					–	VC–C4	Feb	QC
014¶	15.4 (2)	A^+^B^+^, *cdtB^–^*	No	0	NAP4	GB–C1	May	QC
					–	GB–C2	Jan	QC
C	7.7 (1)	A^+^B^+^, *cdtB^–^*	No	–	–	GB–C1	Jan	ON
F	7.7 (1)	A^-^B^+^, *cdtB^–^*	No	VIII	NAP9	GB–C2	Jan	QC
H	7.7(1)	A^+^B^+^, *cdtB^-^*	No	0	Unnamed	GB – C1	Jun	QC
K	7.7(1)	A^+^B^+^, *cdtB*^+^	18 bp	III	NAP1-r	VC–C2	Aug	QC

*PFGE, pulsed-field gel electrophoresis; NA, not amplified because it lacks pathogenicity locus; VC, veal chops; GB, ground beef; C1, rinsate/TCDMNB; C2, meat/TCDMNB; C3, meat/TCDMNB duplicate; C4, meat/TCFFB; QC, Québec; ON, Ontario; SK, Saskatchewan; –, not performed. †Bidet’s PCR ribotyping method (*9*); 077 and 014; representative ribotypes with international nomenclatures assigned by Dr Jon Brazier, University of Wales, Wales, in a previous study (*11*). M26, non-toxigenic Canadian meat ribotype lacking pathogenicity locus (pers. com., M. Rupnik, University of Maribor, Slovenia) (*5*). ‡A, B; *tcdA* and *tcdB* genes. *cdtB,* binding segment of binary toxin; – and + superscripts indicate absence or presence of the gene. *tcdC* gene: no deletions (≈345 bp); 18 bp, deletion type B/C (*8*). §Nomenclature at the Centers for Disease and Control and Prevention, Atlanta, GA, USA. NAP1, North America PFGE type 1. ¶Meat PCR ribotypes matching concurrent local and international human ribotypes (*2*,*3*). Note that 28 *C. difficile* isolates initially identified were grouped into 15 strains based on molecular characteristics and source of origin; 2 meat samples simultaneously harbored 2 strains.

**Figure 2:**
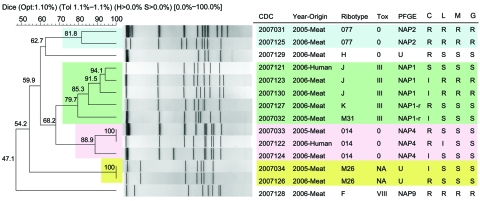
Pulsed-field gel electrophoresis (PFGE)–*SmaI* dendogram of *Clostridium difficile* isolates of meat and human origin in Canada. Representative PCR ribotypes 077, 014, M31, and M26 are of meat origin from 2005 (*4*,*11*). PCR ribotype designations are described in Table 2. Note the genetic similarity (94.1%–100%) and antimicrobial resistance profiles between human and meat isolates, especially PCR ribotypes 014 and J. Also note the genetic similarity (81.8%–100%) between meat isolates from 2005 and 2006 for multidrug-resistant epidemic PCR ribotype 077, clindamycin-variable, PCR ribotype 014, and nontoxigenic PCR ribotype M26. Resistance to all 4 antimicrobial drugs was observed in meat isolates of ribotypes 077 and F, which also yielded the highest level of clindamycin resistance (>256 µL/mL; breakpoint: >6 µL/mL). The breakpoints for moxifloxacin (*12*) were also used for levofloxacin and gatifloxacin. R (resistant), S (susceptible), and I (intermediate) represent antimicrobial profiles. CDC, Centers for Disease Control and Prevention; NAP, North America PFGE type; NAP1-r, NAP-related strain; Tox, toxinotyping nomenclature (M. Rupnik, Maribor, Slovenia); U, unnamed.

PFGE confirmed that selected meat and human PCR ribotypes were identical (Figure 2). Fluoroquinolone and clindamycin resistance was common (41.6%–58.3%) among isolates tested (Figure 2).

## Conclusions

In contrast to our first study (*4*), this study evaluated the genetic diversity of *C. difficile* in retail meats in a large area of Canada and tested 1–2 samples per store to prevent clustering. Thus, the overall prevalence observed (6.1%) was lower than that of previous studies in Canada (20%) (*5*) and the United States (42%) (*13*). Although different sampling and culture methods may account for the different prevalences, taken altogether, these studies support recent concerns regarding food safety.

Duplicate cultures, irrespective of method, could yield higher rates of *C. difficile* recovery from meat. However, the sensitivity of duplicate testing of meat is still suboptimal (46.2%–61.5%) compared with the sensitivity reached by one of our methods (*4*) in human stool samples (>95%) (*6*). Suboptimal performance might be due to reduced culture selectivity and nonhomogeneous distribution or a low number of spores.

In addition to cross-contamination at slaughter and during processing, it is possible that contamination of muscle tissue with *C. difficile* spores occurs preharvest. In horses, *Clostridium* spores have been recovered from muscle tissue in healthy horses (*14*), and a recent muscle sample yielded *C. difficile* in a healthy cow (unpub. data). Translocation from the intestines and deposition of dormant spores in muscle are reasonable assumptions that need investigation.

The increased recovery of *C. difficile* from meat in winter suggests that a seasonal component might exist. This component is currently uncertain, but a possible epidemiologic link between this observation and the seasonality observed in human disease (*15*) and the high rate of *C. difficile* toxins in calves in winter (*11*) requires further elucidation.

The *C. difficile* genotypes identified in this and other studies (especially the NAP1 clone and PCR ribotypes 077 and 014) (*3*,*4*,*11*) provide further molecular evidence that spore dissemination through foods should be considered. Although ingestion of spores does not necessarily imply infection, this study supports the potential for foodborne transmissibility and raises questions about possible seasonality.
